# Effect of Self-developed Ye’an Analgetic Decoction/Jiawei Shaoyao Gancao Decoction Combined with Tramadol on TCM Symptom Scores and RLS Severity of patients with Restless Legs Syndrome

**DOI:** 10.12669/pjms.40.5.8400

**Published:** 2024

**Authors:** Bowen Zhou, Wei Li, Yajun Li, Dongkai Sun, Xin Du

**Affiliations:** 1 Bowen Zhou, Orthopedics of Traditional Chinese Medicine, Baoding No.1 Central Hospital, Baoding, Hebei, 071000, China; 2 Wei Li, Orthopedics of Traditional Chinese Medicine, Baoding No.1 Central Hospital, Baoding, Hebei, 071000, China; 3 Yajun Li, Orthopedics of Traditional Chinese Medicine, Baoding No.1 Central Hospital, Baoding, Hebei, 071000, China; 4 Dongkai Sun, Orthopedics of Traditional Chinese Medicine, Baoding No.1 Central Hospital, Baoding, Hebei, 071000, China; 5 Xin Du, Department of Gynaecology, Baoding Hospital of Traditional Chinese Medicine, Baoding, Hebei, 071000, China

**Keywords:** Restless leg syndrome, Self-developed Ye’an Analgetic Decoction, Jiawei Shaoyao Gancao Decoction, Traditional Chinese medicine symptoms

## Abstract

**Objective:**

To investigate the effect of self-developed Ye’an Analgetic Decoction/Jiawei Shaoyao Gancao Decoction on Traditional Chinese Medicine (TCM) symptom scores and RLS Severity of patients with restless legs syndrome (RLS).

**Methods:**

This was a clinical comparative study. Eighty patients with RLS admitted to Baoding No.1 Central Hospital from January 2022 to December 2022 were randomly divided into observation group and control group(n=40). Patients in the control group were given basic and oral tramadol treatment, while those in the observation group were given self-developed Ye’an Analgetic Decoction/Jiawei Shaoyao Gancao Decoction based on the treatment in the control group. The differences of TCM symptom scores, RLS severity (IRLS), quality of life (QOL-RLS), sleep quality (PSQI) and clinical efficacy between the two groups were compared.

**Results:**

Before treatment, no statistically significant differences were observed in the TCM symptom scores, IRLS scores, QOL-RLS scores and PSQI scores between the two groups (p>0.05). After treatment, the above scores decreased significantly in both groups, with a higher degree of decrease in the observation group than in the control group, indicating statistically significant differences (p<0.05). The QOL-RLS scores were significantly higher in the observation group than in the control group, with a statistically significant difference (p<0.05). The overall response rate in the observation group was 95.00%, which was higher than that in the control group (80.00%), with a statistically significant difference (p<0.05).

**Conclusion:**

Self-developed Ye’an Analgetic Decoction/Jiawei Shaoyao Gancao Decoction leads to numerous benefits in the treatment of RLS, such as obviously ameliorating patients’ clinical symptoms, reducing RLS severity, and improving their quality of life and sleep quality.

## INTRODUCTION

Restless legs syndrome (RLS) refers to a chronic neurological sensorimotor disorder in which the main symptoms are a strong desire for limb movement and abnormal sensation during sleep or quiet. The symptoms are most common in the lower limbs and can also involve the upper limbs, which are often accompanied by unbearable discomfort such as acupuncture, itching, creeping sensation, soreness and pain.^1^ RLS was first described by Tomas Willis, a British doctor, in 1685, and was named and described in more detail by Karl Axel Ekbom who was a Swedish doctor in 1944, so RLS is also called Willi-Ekbom Disease (WED).^2^ RLS makes inroads on 1.2%-15% of the population and is common in Europe and the United States, with a lower prevalence in Asian countries. Moreover, its prevalence differs significantly between men and women, with a significantly higher prevalence in women than in men, and it will gradually increase with age. RLS is common in middle-aged and elderly people in China.^3^-^5^ There are many options available for the treatment of RLS. In Western medicine, drugs are mainly used to relieve or control clinical symptoms^6^, but there are drawbacks such as drug resistance, dependence and side effects that can easily occur with long-term medication.

In contrast, Traditional Chinese Medicine (TCM) takes the overall concept and dialectical treatment as the guiding ideology. Currently, RLS is classified as “arthralgia” in TCM, with its pathogenesis being deficiency of liver and kidney and deficiency of qi and blood. To address this, Jiawei Shaoyao Gancao Decoction can be used for its effects of nourishing yin, nourishing liver and softening tendons, dredging channels and activating collaterals and balancing qi and blood. In this study, the clinical effect of self-developed Ye’an Analgetic Decoction/Jiawei Shaoyao Gancao Decoction on RLS and its influence on TCM symptom scores and RLS severity were investigated.

## METHODS

This was a clinical comparative study. Eighty patients with RLS who met the inclusion and exclusion criteria in Baoding No.1 Central Hospital from January 2022 to December 2022 were selected as subjects and were randomly divided into two groups: the observation group and the control group, with 40 cases in each group.

### Ethical Approval

The study was approved by the Institutional Ethics Committee of Baoding No.1 Central Hospital (No.: [2021]036; Date: November 22,2021), and written informed consent was obtained from all participants.

### Inclusion criteria:

- Patients aged 18-80 years;
- Patients who met the diagnostic criteria for RLS of the “Consensus of Diagnostic Criteria for Restless Legs Syndrome” formulated by the International Restless Legs Syndrome Study Group (IRLSSG) in 2014^7^;
- Patients with no obvious positive signs in physical examination of nervous system and no other serious organic diseases in imaging examination;
- Patients with complete information and valid data;
- Patients who gave informed consent, voluntarily participated in this study, and were able to cooperate with the treatment and fill in the scale.

### Exclusion criteria:

- Patients who did not cooperate with treatment and completed the scale;
- Patients with mental illness or severe organ dysfunction;
- Patients with insomnia and anxiety caused by organic diseases or psychotropic drugs;
- Patients with incomplete data;
- Patients during pregnancy or lactation.

### Basic treatment

Patients were given sleep hygiene, behavioral therapy and elimination of substances that may exacerbate RLS symptoms, such as smoking, alcohol consumption and certain drugs. Patients in the control group were given oral tramadol hydrochloride tablets 50 mg each time, three times/day on the basis of the basic treatment. Those in the observation group were treated with self-developed Ye’an Analgetic Decoction/Jiawei Shaoyao Gancao Decoction on top of the treatment in the control group. The composition of the formula was as follows: 30- g of Radix Paeoniae Alba, 10- g of Radix Glycyrrhizae Preparata, 30- g of Radix Astragali, 20- g of Fructus Chaenomelis, 15- g of Caulis Spatholobi, 15- g of Radix Angelicae Sinensis, 15- g of Radix Achyranthis Bidentatae, 15- g of Radix Salviae Miltiorrhizae, 10 g of Flos Carthami, 10- g of parched Semen Persicae, 10- g of Lumbricus and 6- g of Ramulus Cinnamomi, which were decocted in water to 300 ml, and taken warm in the morning and evening for eight weeks. All Chinese medicines were decocted by the Chinese pharmacy in our hospital.

### Observation indicators

Follow-up in the form of telephone and outpatient clinics was carried out for more than three months from the time of patients’ first hospital discharge as the starting point. *TCM symptom score:* the main symptom (numbness of limbs, weakness of limbs, soreness and pain) and the secondary symptom (insomnia and dreaminess, feverish sensation of five nerves and dry mouth and throat) were given zero, one, two and three points respectively according to the four degrees of symptoms: none, mild, moderate and severe, respectively, with higher scores indicating more severe symptoms.

### RLS severity

The RLS severity was assessed by the International Restless Legs Scale (IRLS), which included 10 questions, each followed by five options, namely, zero for “no discomfort”, one for “mild”, two for “moderate”, three for “severe” and four for “extremely severe”. Out of 40 points, zero means no restless leg symptoms, 1-10 means mild, 11-20 means moderate, 21-30 means severe, and 31-40 means extremely severe.

### Quality of life

The quality of life of RLS patients was assessed using the Quality of Life in Restless Legs Syndrome Questionnaire (QOL-RLS). The questionnaire contained 18 questions, each of which scored 1-5 points, ranging from the mildest to the most severe. Using the formula of (actual score - lowest possible score)/range of possible scores ×100, the scores were transformed into 0-100, with lower scores indicating lower quality of life of patients and higher degree of being affected by RLS.

### Sleep quality

The sleep status of the patients was assessed by Pittsburgh Sleep Quality Index Table (PSQI), which consisted of seven parts, with a total score ranging from 0 to 21. The higher the score, the worse the sleep quality. A total PSQI score of ≥8 indicates the presence of sleep disorders.

### Clinical efficacy

Cured: disappearance of discomfort and normal sleep; Markedly effective: significant reduction of discomfort, which can be relieved by itself without activity and does not affect sleep; Effective: relief of discomfort but still requires activity to relieve, with poor sleep; Ineffective: no improvement in symptoms. Mean follow up period was four months.

### Statistical analysis

All the data in this study were statistically analyzed by SPSS 21.0 software. The measurement data were expressed by mean standard deviation (*χ̅*±S), and *t* test was used for comparison between groups. Counting data were expressed by the percentage of cases [n (%)], and χ^2^ test or Fisher’s exact probability was used for comparison between groups. Rank sum test was used for grade data, with p<0.05 indicating a statistically significant difference.

## RESULTS

No statistically significant differences were observed in the basic data between the two groups, which were comparable (p>0.05). See Table-I for details. Before treatment, no statistically significant differences were observed in the TCM symptom scores between the two groups (p>0.05). After treatment, the TCM symptoms scores decreased significantly in both groups, with a higher degree of decrease in the observation group than in the control group, indicating a statistically significant difference (p<0.05). Table-II .

**Table-I:** Comparison of general information between the two groups.

Item	Observation group (n=40)	Control group (n=40)	t/² value	p-value
Age (years)	60.75±7.79	57.93±8.63	1.537	0.128
Gender (male/female, n)	22/18	24/16	0.205	0.651
Course of disease (y)	13.13±1.67	12.95±1.58	0.481	0.632

**Table-II:** Comparison of TCM symptom scores between the two groups before and after treatment (*χ̅*±*S*, points).

Time	Group	Limb numbness	Limb fatigue	Soreness and pain	Insomnia and dreaminess	Dysphoria with feverish sensation in chest	Dry mouth and throat
Before treatment	Observation group	2.60±0.50*	2.65±0.48*	2.78±0.42*	2.73±0.45*	2.70±0.46*	2.65±0.48*
	Control group	2.70±0.46	2.75±0.44	2.85±0.36	2.65±0.48	2.68±0.47	2.78±0.42
After treatment	Observation group	0.98±0.70^※,△^	0.73±0.64^※,△^	0.75±0.44^※,△^	0.80±0.52^※,△^	0.83±0.50^※,△^	0.80±0.65^※,△^
	Control group	1.40±0.63^△^	1.30±0.56^△^	1.13±0.61^△^	1.13±0.69^△^	1.18±0.68^△^	1.23±0.58^△^

***Note:***

* indicates p>0.05 compared with the control group;

※ indicates p<0.05 compared with the control group of the same period,

△ indicates p<0.05 compared with the pre-treatment group.

Before treatment, no statistically significant differences were observed in the IRLS scores between the two groups (p>0.05). After treatment, the IRLS scores decreased significantly in both groups, with a higher degree of decrease in the obser Table-III.

**Table-III:** Comparison of IRLS scores between the two groups before and after treatment (*χ̅*±*S*, points).

Group	Before treatment	After treatment
Observation group (n=40)	27.23±1.00	11.30±0.97^※,△^
Control group (n=40)	27.60±1.22	16.53±1.20^△^

***Note:***

※ indicates p<0.05 compared with the control group,

△ indicates p<0.05 compared with that before treatment.

Before treatment, no statistically significant differences were observed in the QOL-RLS scores between the two groups (p>0.05). After treatment, the QOL-RLS scores decreased significantly in both groups, with a higher degree of decrease in the observation group than in the control group, indicating a statistically significant difference (p>0.05). Table-IV

**Table-IV:** Comparison of QOL-RLS scores between the two groups before and after treatment (*χ̅*±*S*, points).

Group	Before treatment	After treatment
Observation group (n=40)	56.93±1.38	73.05±2.82^※,△^
Control group (n=40)	57.33±1.44	67.80±2.38^△^

***Note:***

※ indicates p<0.05 compared with the control group,

△ indicates p<0.05 compared with that before treatment.

Before treatment, no statistically significant differences were observed in the PSQI scores between the two groups (p>0.05). After treatment, the PSQI scores decreased significantly in both groups, with a higher degree of decrease in the obser Table-V

**Table-V:** Comparison of PSQI scores between the two groups before and after treatment (*χ̅*±*S*, points).

Group	Before treatment	After treatment
Observation group (n=40)	14.93±1.19	5.95±0.88^※,△^
Control group (n=40)	15.25±1.08	7.53±0.96^△^

***Note:***

※ indicates p<0.05 compared with the control group,

△ indicates p<0.05 compared with that before treatment.

After treatment, the symptoms of the two groups were improved to varying degrees. The overall response rate of the patients in the observation group was 95.00%, which was higher than that in the control group (80.00%), with a statistically significant difference (p>0.05). Table-VI

**Table-VI:** Comparison of the clinical efficacy between the two groups [n(%)].

Group	n	Cured	Markedly effective	Effective	Ineffective	overall response rate (%)
Observation group	40	14 (35.00)	20 (50.00)	4 (10.00)	2 (5.00)	38 (95.00)
Control group	40	11 (27.50)	17 (42.50)	3 (7.50)	8 (20.00)	32 (80.00)
χ^2^ value						4.114
p value						0.043

## DISCUSSION

It was shown in this study that the TCM syndrome scores and IRLS scores of patients in the two groups were significantly decreased compared with those before treatment. The degree of decrease in the observation group was better than that in the control group (p<0.05), indicating that the observation group had better effects in improving the clinical symptoms such as numbness of limbs, weakness of limbs, soreness and pain, insomnia and dreaminess, which was related to the mechanism of nourishing blood and promoting blood circulation, relieving pain and removing blood stasis and dredging collaterals in Jiawei Shaoyao Gancao Decoction. After treatment, the QOL-RLS scores of patients in both groups were higher than those before treatment, while the PSQI scores were significantly lower than those before treatment. Moreover, the improvement of QOL-RLS scores and PSQI scores in the observation group was better than that in the control group (p<0.05). The results showed that the quality of life and sleep of patients with RLS were significantly improved after treatment with Jiawei Shaoyao Gancao Decoction, and its mechanism was related to that Jiawei Shaoyao Gancao Decoction could significantly relieve RLS symptoms, and the RLS symptoms of patients were improved. After treatment, the overall response rate of the patients in the observation group was higher than that in the control group (p<0.05), which further showed that the application of Jiawei Shaoyao Gancao Decoction in the treatment of RLS could effectively relieve the clinical symptoms of patients, regulate their sleep disorders, improve the relationship between them and promote benign recovery.

RLS is a sensory dyskinesia disease and a common disease of the clinical nervous system.^8^ Its clinical symptoms are often separated from physical signs, with the most prominent clinical manifestation being the strange sensation in the deep part of one or both limbs, such as unbearable acid, swelling, numbness or insect bite, ant drilling, or pain such as knife cutting, over-current and tearing. Patients suffering from RLS are unable to relieve the discomfort by resting, forcing them to relieve after activities such as patting, moving the affected limb or walking, which often leads to sleep disorders and anxiety. RLS discomfort symptoms interact with sleep and anxiety to form a vicious circle, which seriously affects patients’ normal work and life.^9^-^12^ Despite numerous clinical studies on the etiology and pathogenesis of RLS, none of them are clear and definitive. It is generally believed that RLS may have a close bearing on dopamine system dysfunction, peripheral neuropathy, iron deficiency, bone diseases and genetic factors.^13^-^15^ RLS has no exact TCM disease name, but its symptoms are similar to “blood arthralgia” and “sore leg” described in Synopsis of the Golden Chamber and Neijing. At present, most TCM scholars classify it as “arthralgia syndrome”, which is marked by three evils of wind-cold-dampness, stagnation of qi-flowing, and dystrophy of meridians. The original symptoms are deficiency of liver and kidney, deficiency of qi and blood and yin and yang, and the treatment is to regulate liver, nourish blood and soothe the nerves.^16^

Shaoyao Gancao Decoction is a classic prescription from Treatise on Febrile Diseases to treat “foot contracture”^17^,^18^, which has the effect of “restoring its yin” if the syncope becomes warmer enough, it is even more like Shaoyao Gancao Decoction. The prescription is composed of Paeonia lactiflora and Radix Glycyrrhizae Moxibustion, and the Paeonia lactiflora is sour and cold, which softens the liver and relieves pain, nourishes blood and astringes yin. In this study, the self-developed Ye’an Analgetic Decoction is composed of Radix Astragali, Fructus Chaenomelis, Caulis Spatholobi, Radix Angelicae Sinensis, Radix Achyranthis Bidentatae, Radix Salviae Miltiorrhizae, Flos Carthami, parched Semen Persicae, Lumbricus and Ramulus Cinnamomi. Caulis Spatholobi can dredge collaterals, relieve pain, enrich blood and promote blood circulation; Angelica regulates menstruation, relieves pain, enriches blood and promotes blood circulation; Carthamus tinctorius has the functions of removing blood stasis, relieving pain, dredging channels and promoting blood circulation; Peach kernel can remove blood stasis and promote blood circulation; Lumbricus can relieve pain and dredge collaterals; Astragalus qi and blood; Achyranthes bidentata, Papaya, Salvia Miltiorrhiza and Ramulus Cinnamomi dredge meridians and reach limbs. The combination of all kinds of drugs has the effects of nourishing blood and activating blood, nourishing yin and softening liver, removing blood stasis and dredging collaterals.^19^-^21^

### Limitations

It includes fewer observation cases with short follow-up time, so it is necessary to expand the sample size and extend the follow-up time to further evaluate its clinical effect. In addition, only the clinical efficacy was observed in this study, which led to the observation indexes being subjectively influenced by patients and the indexes being difficult to quantify, so further studies are still needed to improve them in the future.

## CONCLUSION

Self-developed Ye’an Analgetic Decoction/Jiawei Shaoyao Gancao Decoction leads to favorable clinical results in the treatment of RLS, such as effectively ameliorating the clinical symptoms of RLS patients, reducing RLS severity, and improving their quality of life and sleep, which is worthy of clinical promotion.

### Authors’ Contributions:

**BZ** and **XD** carried out the studies, participated in collecting data, drafted the manuscript, are responsible, accountable for the accuracy and integrity of the work.

**WL** and **YL** performed the statistical analysis and participated in its design.

**DS** participated in acquisition, analysis, or interpretation of data and draft the manuscript.

All authors read and approved the final manuscript.
